# Virulence genes detection of *Salmonella* serovars isolated from pork and slaughterhouse environment in Ahmedabad, Gujarat

**DOI:** 10.14202/vetworld.2015.121-124

**Published:** 2015-01-30

**Authors:** J. H. Chaudhary, J. B. Nayak, M. N. Brahmbhatt, P. P. Makwana

**Affiliations:** Department of Veterinary Public Health, College of Veterinary Science and Animal Husbandry, AAU, Anand - 388 001, Gujarat, India

**Keywords:** pork, *Salmonella*, slaughterhouse environment, virulence genes

## Abstract

**Aim:**

The aim was to detect virulence gene associated with the *Salmonella* serovars isolated from pork and Slaughterhouse environment.

**Materials and Methods:**

*Salmonella* isolates (n=37) used in this study were isolated from 270 pork and slaughter house environmental samples collected from the Ahmedabad Municipal Corporation Slaughter House, Ahmedabad, Gujarat, India. *Salmonella* serovars were isolated and identified as per BAM USFDA method and serotyped at National *Salmonella* and *Escherichia* Centre, Central Research Institute, Kasauli (Himachal Pradesh, India). Polymerase chain reaction technique was used for detection of five genes, namely *inv*A, *spv*R, *spv*C, *fim*A and *stn* among different serovars of *Salmonella*.

**Results:**

Out of a total of 270 samples, 37 (13.70%) *Salmonella* were isolated with two serovars, namely Enteritidis and Typhimurium. All *Salmonella* serovars produced 284 bp *inv*A gene, 84 bp *fim*A and 260 bp amplicon for enterotoxin (*stn*) gene whereas 30 isolates possessed 310 bp *spv*R gene, but no isolate possessed *spv*C gene.

**Conclusion:**

Presence of *inv*A, *fim*A and *stn* gene in all isolates shows that they are the specific targets for *Salmonella* identification and are capable of producing gastroenteric illness to humans, whereas 20 Typhimurium serovars and 10 Enteritidis serovars can able to produce systemic infection.

## Introduction

Pork is one of the most widely eaten meats in the world, accounting for about 38% of meat production worldwide, although consumption varies widely from place to place [1]. Most of the pork consumer’s peoples are from tribal areas and pork is mainly consumed in the northeastern states of India. The present production of meat in India is estimated at 6.27 million tons in 2013 [2], which are 2.21% of the world’s meat production. The meat production has increased from 764,000 tons in 1970-71 to 6.27 million tons in 2010 in India, which is 2.21% of the world’s meat production. The contribution of meat from a pig is 5.31% [2]. According to the Food and Agriculture Organization of the United Nations, world’s pork production reached 114.2 million tons in 2012. Asia is the principal region, accounting for almost 60% of world pig meat production, World meat production is anticipated to expand modestly in 2013 to reach 308.3 million tons, an increase of 4.2 million tones or 1.4%, compared with 2012 [3].

Food safety hazards caused by food-borne pathogens such as *Salmonella* remain a major problem for the food industry. Salmonellosis is an important health problem and a major challenge worldwide having greater significance in developing countries [4]. Pork and pork products are recognized as an important source of human salmonellosis [5]. *Salmonella* is an important cause of food-borne (alimentary) health problems in humans [6]. The risk of *Salmonella* might differ between the production systems, caused by components of the husbandry systems affecting disease development and pathogen shedding or differences in the level of resistance to the pathogen [7]. The increased consumption of pork coupled with the high prevalence of enteropathogens in the swine industry suggests a rise in food-borne illness cases which can lead to human food-borne illness and loss of product shelf-life.

The virulence of *Salmonella* is linked to a combination of chromosomal and plasmid factors. Different genes such as *inv*, *spv*, *fim*A and *stn* have been identified as major virulence genes responsible for salmonellosis. *Salmonella* pathogenicity islands (SPIs) are large gene cassettes within the *Salmonella* chromosome that encode determinants responsible for establishing specific interactions with the host, and are required for bacterial virulence in a given animal like other pathogenicity islands. More than 20 SPIs have been described [8]. The chromosomally located invasion gene *inv*A codes for a protein in the inner membrane of bacteria that is necessary for invasion of epithelial cells [9]. Whereas, an operon (*spv*RABCD), containing five genes, is present on plasmids commonly associated with some serotypes. One main function of the *spv* operon is to potentiate the systemic spread of the pathogen [10]. The *spv*C is virulence-related gene on the plasmid required for survival within host cell [11]. Some studies have provided evidence that the virulence plasmid plays a significant role in human disease [12]. *Salmonella* induced diarrhea is a complex phenomen on involving several pathogenic mechanisms, including production of enterotoxin. This enterotoxin production is mediated by the *stn* thus it plays a significant role in causing gastroenteritis by producing enterotoxin [13].

The purpose of this study was to evaluate the potential virulence of *Salmonella* isolates from eggs and poultry house environment by detecting the presence of the *inv*A, *spv*R, *spv*C, *fim*A and *stn* virulence genes using the polymerase chain reaction (PCR).

## Materials and Methods

Approximately, a total of 270 samples of pork and slaughterhouse environment will be collected from the Ahmedabad Municipal Corporation Slaughterhouse, Ahmedabad, Gujarat under aseptic precautions. The samples were collected in sterilized polyethylene bags and transported to the departmental P.G. Research Laboratory in an icebox for further processing and microbiological analysis. All the samples collected are shown in Table-1.

**Table-1:** Number of samples collected from different sources for isolation of *Salmonella* spp.

Type of sample	Number of samples
Muscles	30
Tonsils	30
Rectal swabs	30
Intestine	30
Lymph node	30
Water	30
Liver	30
Knife swab	30
Butchers hand swab	30
Total	270

Our study used *Salmonella* isolates (n=37) recovered from pork and Slaughterhouse environmental samples collected from the Ahmedabad Municipal Corporation Slaughterhouse, Ahmedabad, (Gujarat), India. 13 *Salmonella* *enteritidis* and 24 *Salmonella* *typhimurium* *Salmonella* serovars were isolated and identified as per BAM USFDA method [14] and serotyped at National *Salmonella* and Escherichia Centre, Central Research Institute, Kasauli (Himachal Pradesh, India). The DNA of isolates of *Salmonella* was prepared by boiling method. Approximately, loop full of culture was taken in microcentrifuge in 100 µl of sterilized DNAse and RNAse-free milliQ water (Millipore, USA). Then, vortexed and samples were heated at 95°C for 10 min, cell debris was removed by centrifugation and 3 μl of the supernatant was used as a DNA template in PCR reaction mixture. PCR was performed with four sets of primer pairs specific for the invasion gene *inv*A, *spv*R gene, *spv*C gene, *fim*A gene and *stn* gene as shown in Table-2.

**Table-2:** Primer pairs used for virulence characterization of *Salmonella* isolates.

Primer pair target	Primer sequence (5’→3’)	Annealing temp (°C)	Length (bp)	Reference
*inv*A	F: GTG AAA TTA TCG CCA CGT TCG GGC AA R: TCA TCG CAC CGT CAA AGG AAC C	63	284	[15]
*spv*R	F: CAG GTT CCT TCA GTA TCG CA R: TTT GGC CGG AAA TGG TCA GT	57	310	[16]
*spv*C	F: ACT CCT TGC ACA ACC AAA TGC GGA R: TGT CTT CTG CAT TTC GCC ACC ATC A	63	571	[17]
*fim*A	F: CCT TTC TCC ATC GTC CTG AA R: TGG TGT TAT CTG CCT GAC CA	56	85	[18]
*stn*	F: CTT TGG TCG TAA AAT AAG GCG R: TGC CCA AAG CAG AGA GAT TC	55	260	[20]

PCR amplifications were performed in a final volume of 25 µl containing DNA template (3 µl), ×2 PCR Mastermix (MBI Fermentas) (12.5 µl), 10 pmol/µl of each primer (MWG-Biotech AG, Germany) (1 µl) and 5.5 µl nuclease-free water. Amplification for *inv*A gene was carried out as described by Kumar *et al*. [15] with minor modifications. The reaction conditions involved initial denaturation at 94°C for 3 min, followed by 35 cycles of 94°C for 30 s, 63°C for 30 s, and 72°C for 30 s. A final extension of 5 min at 72°C was employed. The amplification for *spv*R gene was carried out similarly by employing standardized annealing temperature. The *fim*A gene fragment was amplified at annealing temperature of 56°C and extension for 30 s. The *spv*C gene fragment was amplified at annealing temperature of 63°C and extension for 1 min. The amplification for *stn* gene was carried out employing same conditions as *inv*A except annealing at 55°C. Amplification products were separated by electrophoresed on 2% agarose gel stained with 5 µg/ml of ethidium bromide with a 100 bp DNA ladder as molecular weight marker.

## Results and Discussion

All 37 *Salmonella* isolates (13 of which belonged to serovar Enteritidis and 24 belonged to Typhimurium) contained the invasion gene *inv*A, other studies having reported similar results [17,21-24], which was expected since the *inv*A is an invasion gene conserved among *Salmonella* serotypes.

Similar to *inv*A gene all isolates produced 260 bp DNA fragment specific for *stn* gene which was in agreement with other authors [25-27]. Thus, all the *Salmonella* isolates were found highly invasive and enterotoxigenic.

The *fim*A gene was detected in all 37 isolate produced 85 bp DNA fragment. Which is similar to that of Naravaneni and Jamil [18,19] and this demonstrated that *fim*A gene has a high degree of sequence conservation among *Salmonella* serovars. This is very useful in the diagnosis of *Salmonella* organisms at the genus level.

**Figure-1:**
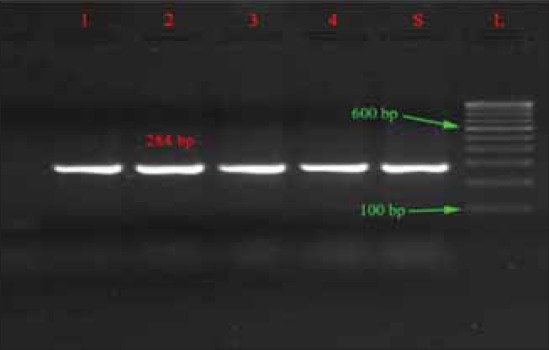
Agarose gel showing polymerase chain reaction amplification products of *inv*A gene (284 bp).

**Figure-2:**
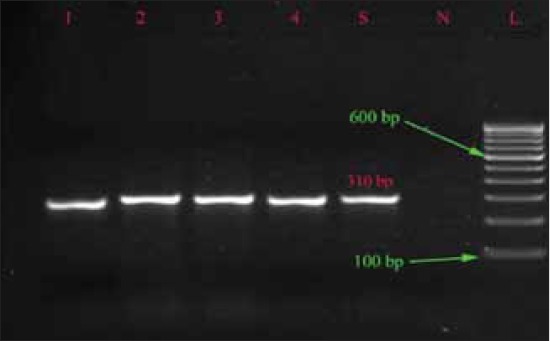
Agarose gel showing polymerase chain reaction amplification products of *spv*R gene (310 bp).

**Figure-3:**
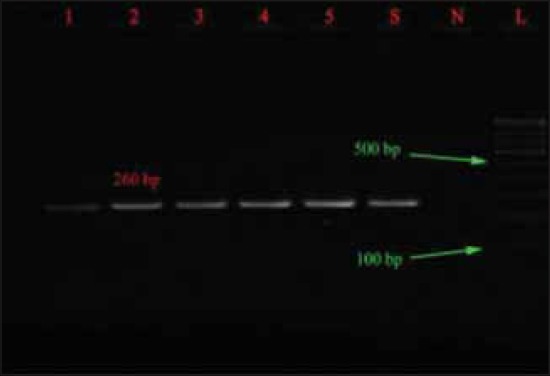
Agarose gel showing polymerase chain reaction amplification products of *stn* gene (260 bp).

**Figure-4:**
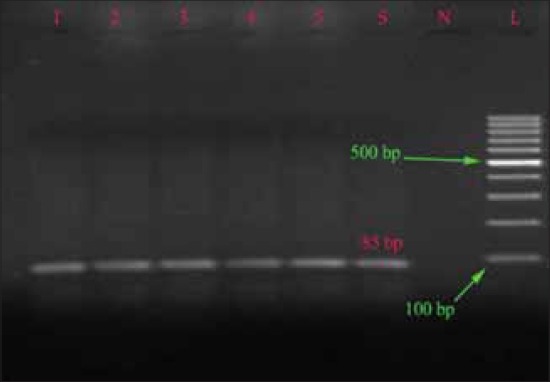
Agarose gel showing polymerase chain reaction amplification products of *fim*A gene (85 bp).

The *spv*R gene was detected in 30 isolates belonged to Typhimurium and Enteritidis, which is similar to that of Araque [23] and this shows that the strains have the plasmid borne virulence characters that have ability to cause the systemic infection while *spv*C was not detected in any isolates, which is in contrast to that of Soto *et al*. [27] who found presence of *spv*C in all the isolates (Table-3). Electrophoreses results of *inv*A, *spv*R, *fim*A and *stn* gene are shown in Figures-1-4, respectively.

**Table-3:** Virulence genes present in different serovars of *Salmonella*.

Serotype	Virulence genes				

	*inv*A	*spv*R	*spv*C	*fim*A	*stn*
Enteritidis (13)	13	10		13	13
Typhimurium (24)	24	30		24	24

## Conclusion

We can conclude that the *inv*A and *stn* genes can be used as specific targets for detection of *Salmonella* as they are conserved among the *Salmonella* irrespective of serotype and plasmid-borne genes (*spv*) are not specific targets for the same.

## Authors’ Contributions

JBN and MNB planned and designed the study. JHC collected and processed samples, the experiment was conducted, and laboratory work was done by JHC and PPM. All authors participated in the preparation of draft of the manuscript and read and approved the final manuscript.
